# Selenium-enriched *Lentinus edodes*: characterization and antidiabetic effects in Wistar rats

**DOI:** 10.3389/ffunb.2026.1866421

**Published:** 2026-07-23

**Authors:** Ivan Milovanovic, Walter Goessler, Saša Vukmirović, Ivan Čapo, Nebojša Stilinović

**Affiliations:** 1 Innovation Centre of the Faculty of Technology and Metallurgy, Belgrade, Serbia; 2 Institute of Chemistry – Analytical Chemistry for Health and Environment, University of Graz, Graz, Austria; 3 Department of Pharmacology, Toxicology, and Clinical Pharmacology, Faculty of Medicine, University of Novi Sad, Novi Sad, Serbia; 4 Department of Histology and Embryology, Faculty of Medicine, University of Novi Sad, Novi Sad, Serbia

**Keywords:** alloxan-induced diabetes, fortified nutrition, Lentinus edodes, selenium speciation, Wistar rats

## Abstract

**Background:**

*Lentinus edodes* is the second most widely cultivated edible mushroom globally. This study aimed to enrich *L. edodes* with selenium, quantify total selenium and its species, and evaluate the *in vivo* antidiabetic activity of selenium-enriched extracts using Wistar rats as the model organism.

**Methods:**

The experiment involved healthy male Wistar strain laboratory rats, divided into 8 groups. Along with general health status, the animals' body weight, blood glucose levels and lipid concentrations as well as selenoprotein P1 and glutathione peroxidase 1 (GPx-1) expression were monitored during the experiment.

**Results:**

The highest selenium accumulation was observed in the first flushes, reaching 90.4 ± 9.0 mg Se kg^-1^. Selenomethionine (Se-Met) was identified as the sole selenium species in both aqueous and enzymatic extracts. The findings reveal subtle variations in the physiological effects of *L. edodes* harvested at different times, particularly regarding body weight, blood glucose, cholesterol, and triglyceride levels, as well as pancreatic tissue health and the expression of selenoprotein P1 and GPx-1 in rats. Selenium-enriched *L. edodes* extracts exhibited notable antidiabetic properties, especially in regulating blood glucose and preserving pancreatic β-cell structure in an alloxan-induced diabetes model. A significant positive correlation was found between selenoprotein P1 levels and total cholesterol (ρ = 0.622; p < 0.001).

**Discussion:**

The findings of this study provide a basis for monitoring the quality and reproducibility of the selenium fortification process. Furthermore, these results suggest that further investigation in additional animal models is warranted to confirm the broader applicability and robustness of this approach.

## Graphical Abstract

**Figure:**
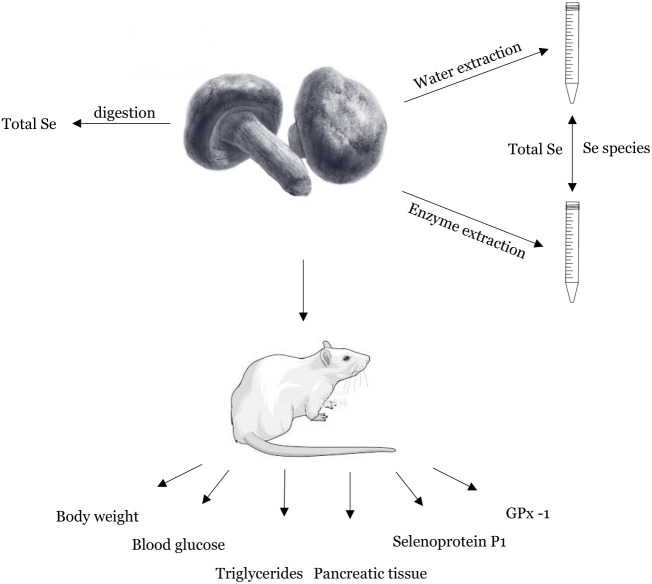

## 1 Introduction

Selenium (Se), a non-metallic trace element, plays a vital role in human and animal health. It is indispensable for maintaining redox homeostasis, immune function, and endocrine regulation. The recognition of selenium as an essential micronutrient dates to the late 1950s, when Schwartz and Foltz (Schwarz and Foltz, 1957) demonstrated its necessity in preventing nutritional deficiency diseases. At the molecular level, selenium exerts its biological effects primarily through its incorporation into selenoproteins, such as glutathione peroxidases, thioredoxin reductases, and selenoprotein P (Labunskyy et al., 2014). These enzymes are integral to the antioxidant defence system, where they participate in the detoxification of reactive oxygen species, the prevention of lipid peroxidation, and the protection of cellular structures, particularly lipid membranes. Glutathione peroxidase (GPx), a prototypical selenium dependent enzyme, catalyzes the reduction of hydrogen peroxide and organic hydroperoxides, utilizing glutathione (GSH) as a reducing agent. This reaction is a central component of cellular antioxidant mechanisms, and its activity is directly influenced by selenium availability (Huang et al., 2018). Epidemiological data suggest that suboptimal selenium status is associated with an increased risk of chronic conditions, including cancer, cardiovascular disease, and immune dysfunction, all of which are partly attributable to impaired selenoprotein synthesis and activity (Schomburg, 2022). The recommended daily intake of selenium for adults ranges between 50 and 200 µg, depending on physiological status and regional dietary patterns (Institute Of Medicine (IOM), 2000).

Beyond its role in cancer prevention and cardiovascular health, selenium has been implicated in the management of various endocrine and metabolic disorders. Clinical and preclinical studies have demonstrated selenium’s potential benefits in thyroid diseases such as Hashimoto’s thyroiditis and autoimmune thyroiditis (Karimi and Omrani, 2019; Hu et al., 2021), in reducing inflammatory complications in sepsis (Kočan et al., 2014) and in the modulation of lipid metabolism (Rayman et al., 2011). With respect to glucose homeostasis, selenium exhibits a dose-dependent, U-shaped biological response. While supplementation at physiological doses may improve glycemic control and insulin sensitivity, excessive or deficient intake has been associated with adverse metabolic outcomes, including hyperglycemia and hyperinsulinemia (Ogawa-Wong et al., 2016; Casanova, 2023). However, inconsistencies across studies owing to geographic variability in selenium intake, methodological differences in dietary assessments, and heterogeneous study designs, underscore the need for more rigorous experimental models and controlled clinical trials (Kohler et al., 2018; Pyrzynska and Sentkowska, 2024).

In this context, the use of selenium-enriched functional foods has emerged as a promising strategy to improve selenium intake in a bioavailable and physiologically tolerable form. Edible mushrooms have attracted particular attention due to their natural capacity to bioaccumulate selenium from cultivation substrates and convert it into organic selenium species, such as selenoamino acids, selenopeptides, and selenoproteins, which exhibit lower toxicity and superior bioavailability compared to inorganic forms (Tsivileva and Perfileva, 2017). Among selenium-accumulating fungal species, *Lentinula edodes* (shiitake), *Agaricus bisporus*, and *Pleurotus* spp. are particularly notable for their capacity to serve as effective carriers of dietary selenium (Witkowska, 2014). Specific selenium-enriched fungal compounds have demonstrated immunomodulatory, antioxidant, and antitumor activities (Hu et al., 2021). Moreover, selenium-enriched mushrooms have shown potential antidiabetic effects by improving glucose metabolism and reducing oxidative stress, a key factor in the development of insulin resistance. Their bioactive compounds, including organic selenium species and polysaccharides, may enhance insulin sensitivity and support the regulation of blood glucose levels (Aramabašić Jovanović et al., 2021).

The present study aimed to develop a standardized preparative and analytical approach for the characterization of selenium compounds in *Lentinus edodes* cultivated under selenium enriched conditions, with respect to health-related and nutritional aspects. Specifically, the objectives were: (i) to characterize selenium species in fruiting bodies harvested at different developmental/harvesting stages, and (ii) to evaluate the biological activity of selenium-enriched *L. edodes* extracts in an alloxan-induced diabetic rat model, with a focus on glycemic control, lipid metabolism, pancreatic tissue integrity, and the expression of selected selenoproteins. The outcomes of this research are intended to support the use of selenium-enriched mushrooms as potential functional foods for metabolic health.

## 2 Materials and methods

### 2.1 Culture collection

The basidiocarp of *Lentinus edodes* (Berk.) Pegler strain ICTMF301 which was used in this study, was obtained from the culture collection of the Innovation Centre of the Faculty of Technology and Metallurgy, Belgrade (ICTMF). The stock culture is maintained on malt agar (MA) and kept in the fungi culture collection of Innovation Centre (Singh and Singh, 2024).

#### 2.1.1 Preparation of seed-inoculum (Spawn)

The spawn is prepared by cooking 100 g of wheat grain for 30 minutes. After cooking, the excess water is drained and supplemented with 2 g of Ca_3_(PO_4_)_2_ and 0.5 g of CaCO_3_ mixed manually, placed in bottles and sterilized in an autoclave at 121 °C for 15 min. Then, each bottle was inoculated with 25 mycelial disks (Ø 0.5 cm) obtained from 7-day-old culture and incubated at 22 ± 2 °C in the dark for 2 weeks (Singh and Singh, 2024).

#### 2.1.2 Fruiting body growth

The mixture of wheat straw (small pieces) and oak dust in ratio 1 + 1 was dipped in distilled water (dH_2_O) for 12 h and centrifuged at 688 g-force for 5 min to remove excess water. Next, 1 kg of substrate was placed into the polypropylene bags and autoclaved at 121 °C for 2 h. The final humidity was 80%. For enrichment, 5 mL of sterile Na_2_SeO_3_ solution, at a concentration of 1.00 g Se L^-1^ were added into bags, respectively, and inoculation with overgrown spawn was performed. A bag without Se was maintained for control purposes. The inoculated bags were incubated at 22 °C in the dark for 4 weeks. Mushrooms were harvested during four flushing times over a total period of 95 days. All harvested fruiting bodies were lyophilized in gamma 1–16 freeze-drying system (Christ, Osterode am Harz, Germany), and ground under liquid nitrogen in a Micro-Dismembrator U (Braun Melsungen, Germany; shaking frequencies 1500 min^-1^) to a fine powder, that was kept at -80 °C until usage (da Silva et al., 2012).

### 2.2 Selenium determination

#### 2.2.1 Total selenium determination along with other trace elements

Dried *L. edodes* fruiting bodies (0.01 g weighed to 0.1 mg) were digested at 90 °C in duplicate with a mixture of 40 µL of nitric acid (p.a., ≥65%) and gallium standard (1000 µg L^-1^). After cooling, the 8 µL of the dilution was applied to the middle of a polished quartz glass slide and dried overnight at 37 °C. A standard sample of human serum served as control in each assay run for quality assessment. The certified standard reference material SNL-1 (Seronorm™ Trace Elements Whole Blood, SERO, Billingstad, Norway) was used for quality control. Finally, the total Se content was determined with total reflection X-ray fluorescence (TXRF) analysis using a benchtop TXRF analyser (S4 T-STAR, Bruker Nano GmbH, Berlin, Germany), as described (Sun et al., 2020).

#### 2.2.2 Extraction of mushroom compounds

Two independent extractions were conducted and compared. For the water extraction, 5.0 mL of water was added to 20 mg (± 0.1 mg) of sample in a 15 mL polypropylene (PP) centrifuge tube. The mixtures were thoroughly homogenized by mechanical shaking in a water bath at 37 °C for 20 h. In the case of enzymatic digestive extraction, 125 µL of proteinase K (10 mg mL^-1^, Thermo Fischer Scientific, Massachusetts, USA) and 5.0 mL of sodium phosphate buffer (100 mM, pH of 7.5, Sigma-Aldrich, Steinheim, Germany) were added to 20 mg of sample in 15 mL PP centrifuge tube. The enzymatic digestion was performed for 20 h in a water bath at 37 °C. Both mixtures were centrifuged at 2000 rpm for 2 min afterwards, and the supernatants were filtered through 0.2 μm syringe filters (Merck, Darmstadt, Germany). To obtain information about the extraction efficiency, aliquots of the extracts were analyzed by TXRF. In order to inhibit proteinase K before cell culture experiments, samples were heated to 95 °C for 10 min (Milovanovic et al., 2024).

#### 2.2.3 Selenium speciation analysis

For Se speciation analysis, the mushroom extracts were diluted 1 + 9 (v/v) with water to avoid an overload of the analytical column and water and enzyme aliquots were subjected to high-performance liquid chromatography (HPLC 1200, Agilent Technologies) coupled to a triple quadrupole inductively plasma mass spectrometry (ICP-MS/MS; Agilent 8900, Agilent Technologies, Waldbronn, Germany). Ion-pairing reversed-phase (IPRP) chromatography was preformed using YMC Triart C18 (150 mm × 2.1 mm, 3 μm; YMC, Tokyo, Japan) as stationary phase, and the mobile phase was an aqueous solution of 0.3% (v/v) heptafluorobutylamin and 3% (v/v) methanol, at pH 4.0 (Lajin and Goessler, 2020). The Se signal was recorded using an element selective ICP-MS/MS detector mode utilizing the octopole reaction system (ORS) with an oxygen flow of 0.4 mL min^-1^ by monitoring the mass transition m/z 78→94 [^78^Se^16^O]^+^. Multi-point calibration was used in a range from 1.0 to 500 μg Se L^-1^ in the quantification modus for each Se species (Se-cystine (Se-Cys2), Se-(methyl) selenocysteine (met-Se-Cys), Se-methionine (Se-Met), selenite (Se (IV)), selenate (Se (VI)) (Sigma- Aldrich, Steinheim, Germany) (Milovanovic et al., 2019).

### 2.3 *In vivo* study

#### 2.3.1 Experimental animals

The experiment involved healthy male Wistar strain laboratory rats, selected randomly, with body weights ranging from 250 to 350 grams. The animals were housed under standard laboratory conditions throughout the experiment in the vivarium of the Department of Pharmacology, Faculty of Medicine, Novi Sad. The air temperature was maintained between 20 and 25 °C, with an air humidity level of 55.0 ± 1.5%. The light-dark cycle alternated every 12 hours. The rats had unrestricted access to standard pelleted food (Veterinary Institute Subotica, Serbia) and tap water during the entire duration of the experiment. The animals’ well-being was assessed daily. All experimental procedures complied with the European Directive (2010/63/EU) on animal experiments and were reviewed and approved by the Ethics Committee for the Protection and Welfare of Experimental Animals at the University of Novi Sad, Serbia and the Ministry of Agriculture, Forestry and Water Economy – Veterinary Directorate (Approval No. 001330935-2024).

#### 2.3.2 Experimental design

A study investigated the antidiabetic potential of *Lentinus edodes* water extracts in 56 rats allocated to eight experimental groups (n = 7 per group), including one healthy control group and seven groups subjected to alloxan-induced diabetes (150 mg kg^-1^, intraperitoneally). Following diabetes induction, blood glucose levels were measured 48 hours post-administration to confirm the development of hyperglycemia. Based on the expected success rate of diabetes induction, 55 animals were ultimately subjected to alloxan administration. Of these, three animals did not develop hyperglycemia (blood glucose ≤ 15 mmol L^-1^). In addition, three animals with the highest glycemic values were excluded before randomization to ensure balanced group allocation. The remaining 49 hyperglycemic animals were randomly assigned to seven diabetic experimental groups (n = 7 per group). Animals that did not meet the inclusion criteria were humanely euthanized in accordance with institutional ethical guidelines by intraperitoneal administration of a 25% urethane solution (0.75 g kg^-1^), followed by cardiopuncture (exsanguination).

Two groups (healthy control - NORM and diabetic control - DIAB) received physiological saline (1 mg kg^-1^, orally) for 7 days, and one diabetic group (METF) was treated with metformin (80 mg kg^-1^). The remaining five diabetic groups were treated with *Lentinus edodes* extracts: one with a non-selenium-enriched extract (LE0) and four with selenium-enriched extracts of different harvesting (LE1-4). All mushroom-treated groups received 100 mg kg^-1^ of the extract, taking care not to exceed 10 µg kg^-1^ of selenium, twice the recommended daily intake (Casanova, 2023). Doses of metformin and mushroom extracts were calculated using the Food and Drug Administration’s Human Equivalent Dose formula for the average person of 70 kg (FDA Guideline).

Along with general health status, the animals’ body weight and blood glucose levels were monitored during the experiment. The treatment lasted seven days, and on the final day, the animals were anaesthetized with an intraperitoneal injection of a 25% urethane solution at a dose of 0.75 g kg^-1^ and sacrificed via cardiopuncture (exsanguination) to obtain blood and internal organs required for subsequent investigations. Tissue samples were collected from the pancreas of the rats, while biochemical parameters, blood glucose levels, and lipid concentrations, were analyzed from the blood samples.

### 2.4 Biochemistry assays

#### 2.4.1 Blood glucose level

The glucose concentration in capillary blood collected from the rats’ tail veins was measured using commercial kits with the Accu-Chek Active device (Roche, Basel, Switzerland). The experimental animals’ starting blood glucose levels were measured 48 hours after the administration of alloxan (Glucose start). The final blood glucose measurement was taken two hours after the last dose (Glucose end) of administered substances.

#### 2.4.2 Blood lipid parameters

The blood samples taken from the animals were left at room temperature to clot. Afterwards, they were centrifuged at 1006, 2 g-force for 10 minutes at 4 °C to obtain serum. Then, the serum concentrations of total cholesterol (mmol L^-1^) and triglycerides (mmol L^-1^) were measured using commercial kits (BioSystems, Costa Brava, Spain). All the tests were run on a Boeco spectrophotometer S-220 UV-Vis (Boeco, Hamburg, Germany).

#### 2.4.3 ELISA analysis

Selenoprotein P1 (SEB809Ra) and glutathione peroxidase 1 (SEA295Ra) levels in rats’ serum were quantified using commercial ELISA kits (Cloud-Clone Corp., Katy, Texas, USA). Before the experiment, all kit components and serum samples were brought to room temperature (20-25 °C). The assays were carried out according to the manufacturer’s instructions. After the last step of both assay procedures, the measurement was immediately conducted at 450 nm on a BioTek 800 TS absorbance reader (Agilent, Santa Clara, California, USA).

### 2.5 Histology analysis

#### 2.5.1 Standard and immunohistochemical staining

The splenic regions of pancreas of each animal were used for histological analysis. After 24h fixation in 4% buffered formalin and appropriate dehydration, the samples were embedded in paraffin and cut at 5 µm on a rotary microtome. Slides were stained on routine hematoxylin-eosin (HE) staining and immunohistochemical staining, including rabbit anti-insulin (Abcam, UK) in a 1 + 199 dilution, and rabbit anti–glucagon (Abcam, UK) in a 1 + 99 dilution, and rabbit anti–Ki67 (Abcam, UK) in a 1 + 199 dilution, using the appropriate visualization system: Mouse and Rabbit Specific HRP/DAB Detection IHC kit, and Mouse and Rabbit Specific HRP/AEC Detection IHC kit (Abcam, UK). Antigen retrieval was performed using a citrate buffer (pH 6.0) in a microwave oven at 850 W for 20 min. Antibodies were applied for 60 min at room temperature with Mayer’s hematoxylin counterstain and finally mounted with DPX medium (Sigma-Aldrich, Germany). Slide analysis and digitalization were performed using a digital microscope VisionTek^®^ (Sakura, Japan).

#### 2.5.2 Morphometrical analysis

Ten immunocytochemically stained sections (40 for insulin and 40 for glucagon) were used for morphometric analysis. Ten islet profiles were randomly selected from each slide/specimen for evaluation, resulting in a total of 800 islets (400 for insulin and 400 for glucagon) examined at a magnification of x630. This approach allowed us to estimate the number of each cell type per islet (Nα and Nβ) using established morphometric formulas (Ilić et al., 2017). Briefly, the numerical density of α- and β-cells was estimated from the number of immunopositive nuclear profiles per unit islet area. Numerical density per unit volume was calculated according to established stereological principles that account for nuclear size and section thickness. The absolute number of α- and β-cells per islet (Nα and Nβ) was obtained by multiplying the numerical density by the estimated islet volume. To calculate the volume density of the endocrine (Vvend) and exocrine portions of the pancreatic tissue (Vvexo), we captured images of six randomly selected fields at a magnification of x400 for each slide (Elayat et al., 1995). The image analysis was performed using a point-counting stereological method implemented in the CAST plugin for ImageJ, and the results were expressed as the fraction of points falling on endocrine or exocrine tissue relative to the number of test points (Ilić et al., 2017).

### 2.6 Statistical analysis

Statistical analysis was performed utilizing IBM SPSS software, version 26.0 (IBM Corp., Armonk, NY, USA). Results were presented as the mean ± standard deviation (SD). Normality of data distribution was assessed using the Shapiro-Wilk test, together with visual inspection of histograms. Homogeneity of variances was evaluated using Levene’s test. Based on these results, either parametric or non-parametric statistical methods were applied where appropriate. Independent or paired samples T-tests were used to compare two groups. One-way analysis of variance (ANOVA) or the Kruskal–Wallis test was applied for comparisons involving more than two groups. Tukey’s test was employed for *post hoc* analysis following ANOVA, whereas the Mann–Whitney U test-applied with or without Bonferroni correction-was used for *post hoc* comparisons in the Kruskal-Wallis analysis. Correlation analysis was conducted using either Pearson’s or Spearman’s correlation coefficient. A p-value of less than 0.05 (p<0.05) was considered statistically significant.

## 3 Results and discussion

### 3.1 Total selenium

**Table 1** shows the observed total Se concentrations of *L. edodes* fruiting bodies (all concentrations are expressed on a dry mass basis) over different harvesting periods. Accumulated Se amounts varied across the range of 23.3-90.4 mg kg^-1^. The Se concentrations in *L. edodes* fruiting bodies gradually decreased with increasing selenium application time. In the control samples, Se was below the detection limit.

**Table 1:** Se concentrations, extraction efficiency (extracted/total Se) in *L. edodes* by using TXRF analyzer.

		Total Se [mg kg^-1^]		Extracted Se [mg kg^-1^]		Extraction efficiency [%]		Column recovery [%]	
Species	Harvesting	Na_2_SeO_3_	Control	Water	Enzyme	Water	Enzyme	Water	Enzyme
*Lentinus edodes*	1^st^	90.4 ± 9.0^*^	n.d	65.8^**^	82.3	72.8	91.1	32.9	34.1
	2^nd^	40.7 ± 0.6		31.2	30.7	76.6	75.4	36.3	34.7
	3^rd^	35.0 ± 1.0		27.6	33.7	78.8	96.2	38.0	27.2
	4^th^	23.3 ± 2.2		20.1	19.4	86.4	83.2	40.7	41.9

*Variations are given as a single standard deviation.

**Data represent mean value of two individual samples.

Previous studies displayed strong ability of cultivated *L. edodes* to accumulate exogenous Se which strictly depends on growing condition. Thus, Zhou et al. (2018) showed absorption rates of 5.91 - 68.2 mg Se kg^-1^ at different Se treatments and harvesting stages. Similar observation reported Gergely and colleagues (Gergely et al., 2006) a total Se of 46.0 mg kg^-1^ in *L. edodes*. Su and colleagues (Su et al., 2024) as well as Assunção and others (Assunção et al., 2014) reported a total Se of 121 mg kg^-1^ and 170 mg kg^-1^ in *L. edodes*. In the study of Yoshida and colleagues (Yoshida et al., 2005) and Ogra et al. (2004) the Se content in Se-enriched shiitake was even much higher 408 mg kg^-1^ and 356 mg kg^-1^. Opposite results might be explained due to the difference in the application time of applied Se. Namely, the reason of added sodium selenite after or before mycelia fully overgrown substrates affect the higher accumulation of Se in mushroom. In the early stage of incubation, mycelia absorbed a large amount of Se from the substrates, and in the first harvesting phase, a total Se reached a maximum. Afterwards, in the middle stage, short time of fructification is the main cause for which Se concentrations declined gradually in *L. edodes* (Zhou et al., 2018). On the other hand, the length of interval time between harvesting cycles may lead to the higher Se content ration in mushroom. Similar to that observed with *Pleurotus* and *Ganoderma* species in case where Se concentrations at the second flush were higher than those from the first one (da Silva et al., 2012; Milovanovic et al., 2024). The difference in the Se bioavailability between species could be another reason for the discrepancy in changes of Se concentration from the first and latest flushes. In addition, based on the Se content in *L. edodes* fruiting bodies, Na_2_SeO_3_ seems to be good choice to supply the amount of Se recommended for adults (Institute Of Medicine (IOM), 2000).

### 3.2 Extraction efficiencies

Extraction efficiencies of the *L. edodes* fruiting bodies from different harvest times were summarized in **Table 1**. Extraction of Se was highest from the first flush as compared to the other flushes in both treatments; enzymatic digestions proved slightly more efficient than water-based treatment especially in the first harvest stage i.e. 82.3% versus 65.8%, but no significant difference in other harvesting treatment. The extraction efficiency rates were quite good and ranged from 72.8 - 96.2% which indicates water/enzyme extractions methods were suitable for Se extraction (**Table 1**).

To release Se from mushroom, we used two commonly used approaches. Water extraction for the water-soluble and non-protein bound amino-acids, but the extraction efficiency is low (Gosetti et al., 2007), and enzymatic hydrolysis extraction which has several advantages including high extraction rates and minimized Se-speciation changes (Grijalba et al., 2017). The use of protease K presents a popular and logical approach for Se extractions in *L. edodes*. This notion is in agreement with similar observations. Thus, Yoshida and colleagues (Yoshida et al., 2005) recorded high extraction rate comparing enzyme 83.2% versus HCl 10.2%; 57.5% and 60.6% extracted Se in *L. edodes* fruiting body (Gergely et al., 2006); and more than 88% of extraction yield from all flushes cycle (Zhou et al., 2018). Interestingly, a comparatively small difference between enzymatic and water treatment was observed for Se-exposed *Lentinus edodes* fruiting bodies (77.5 vs 68.0%) (Ogra et al., 2004). When these observations are taken together with the results of the present study, it becomes clear that there is no significant deviation in Se forms (free or bound) in the second, third and fourth flushes, contrary to the first flush. These differences are due to the development stage (time of harvest) of the mushroom.

### 3.3 Selenium speciation

The chromatogram revealed that Se-met was the major Se compound in the fruit bodies of *L. edodes* in all treatments. In addition, other Se standards were not identified; these compounds peaks were below the limit of quantification. Concentrations of Se-Met in the fruit bodies of *L. edodes* harvested at various stages are shown in **Table 2**. The total detected Se-Met varied from 1.2 – 7.2 mg kg^-1^ on a dry mass. Enzyme extraction was better choice for Se-Met quantification than water treatment; the proportion of Se-Met to the total Se content varied from 20-30%. First flush recorded highest Se-Met content in both aliquots; with application time rate Se-Met gradually decreased. Column recovery of Se based on the sum of peaks relative to the total Se determined in the extracts ranged from 27.2 – 41.9%; no significant differences between aliquots and flushing cycle were detected in *L. edodes*.

**Table 2:** Se species concentrations in *L. edodes* by using (IP) HPLC-ICPMS/MS.

		SeMet [mg kg^-1^]		Total Se [mg kg^-1^]	
Species	Harvesting	Water fraction	Enzyme fraction	Water fraction	Enzyme fraction
*Lentinus edodes*	1^st^	1.9^*^	7.2	29.8	30.8
	3^rd^	1.2	4.1	14.8	14.1
	4^th^	–	2.2	13.3	9.5
	2^nd^	–	2.0	9.5	9.8

*Data represent mean value of two individual samples.

wThis result is in agreement with the Se speciation analysis reported elsewhere. Detection of Se-Met as the most abundant selenoamino acid in *L. edodes* is already recorded (Ogra et al., 2004; Yoshida et al., 2005; Gergely et al., 2006; Assunção et al., 2014; Zhou et al., 2018). This observation can be explained due to the mushroom capacity to absorb inorganic Se and convert to less toxic organic form Se-Met which is further incorporated non-specifically into protein in the place of Met because of an inadequate function of aminoacyl tRNA synthetase (Lobanov et al., 2009). or found to be free as a precursor for ethylene synthesis (Milovanovic et al., 2019). In comparison to the total Se and Se-Met in the flushes, it was likely difficult to be organically transformed within the duration of the harvesting stage. In contrast, Se conversion gradually slowed down because of the transformation consumption of the mycelia in the early stage of fructification. Moreover, the combined forms of Se (water-free or protein-bound) in mushrooms and the dynamic changes of Se-Met during the harvesting period may be influenced by the nature of the species itself. Further analysis is needed to confirm this hypothesis.

### 3.4 *In vivo* study

#### 3.4.1 Body weight

As mentioned, all animals were subjected to body weight measurement at the beginning of the experiment. **Table 3** shows that the animals were randomly distributed into groups, so there is no statistically significant difference in the starting (Start weight) body mass value. In contrast, the body mass measured at the end of the trial (End weight) differ significantly because diabetes was induced in 7 out of 8 groups of animals, and the animals received different treatments. To more objectively assess the impact of the treatment on body weight, the difference between the final and initial body weight (Weight change) was calculated. As expected, body weight gain was largest in a group of healthy animals since diabetes is characterized by loss of fat in adipose tissue as well as with catabolism of proteins in muscles, particularly structural proteins, major contributors to body weight (Eluehike and Onoagbe, 2018). A statistically significant difference in body weight change is evident when comparing the value of the group of normoglycemic animals with all other animals. Among experimental groups with induced diabetes, certain weight gain was detected in four out of seven experimental groups, still without statistical significance. In animals treated with *L. edodes* mushroom, as well as with selenium enriched *L. edodes* of the first and third harvest, a decrease in body weight was recorded. A change in body weight was significant when comparing the groups treated with control *L. edodes* and selenium enriched *L. edodes* of the third harvest with the control group of diabetic animals. Although it is known that Se and selenoproteins play an important role in fatty tissue physiology, our data are inconsistent, what is in line with studies of other authors (Tinkov et al., 2020). Our data supports the fact that additional studies are required to further elucidate the interplay between Se and adipose tissue metabolism.

**Table 3:** Rats’ body weight during the experiment (n = 7; mean ± SD).

Group	Start weight (g)	End weight (g)	Weight change
NORM	333 ± 43	418 ± 39	85 ± 6
DIAB	328 ± 35	364 ± 41	35 ± 29^*^
METF	324 ± 46	335 ± 47^*^	11 ± 29^*^
LE0	320 ± 29	310 ± 35^*^	-9 ± 2^*,#^
LE1	310 ± 47	313 ± 51^*^	-8 ± 16
LE2	311 ± 57	333 ± 45^*^	25 ± 45^*^
LE3	318 ± 18	310 ± 29^*^	-7 ± 15^*,#^
LE4	322 ± 37	345 ± 50	23 ± 17^*^

*p < 0.05 versus NORM group; ^#^p < 0.05 versus DIAB group

#### 3.4.2 Carbohydrate metabolism

**Figure 1:**
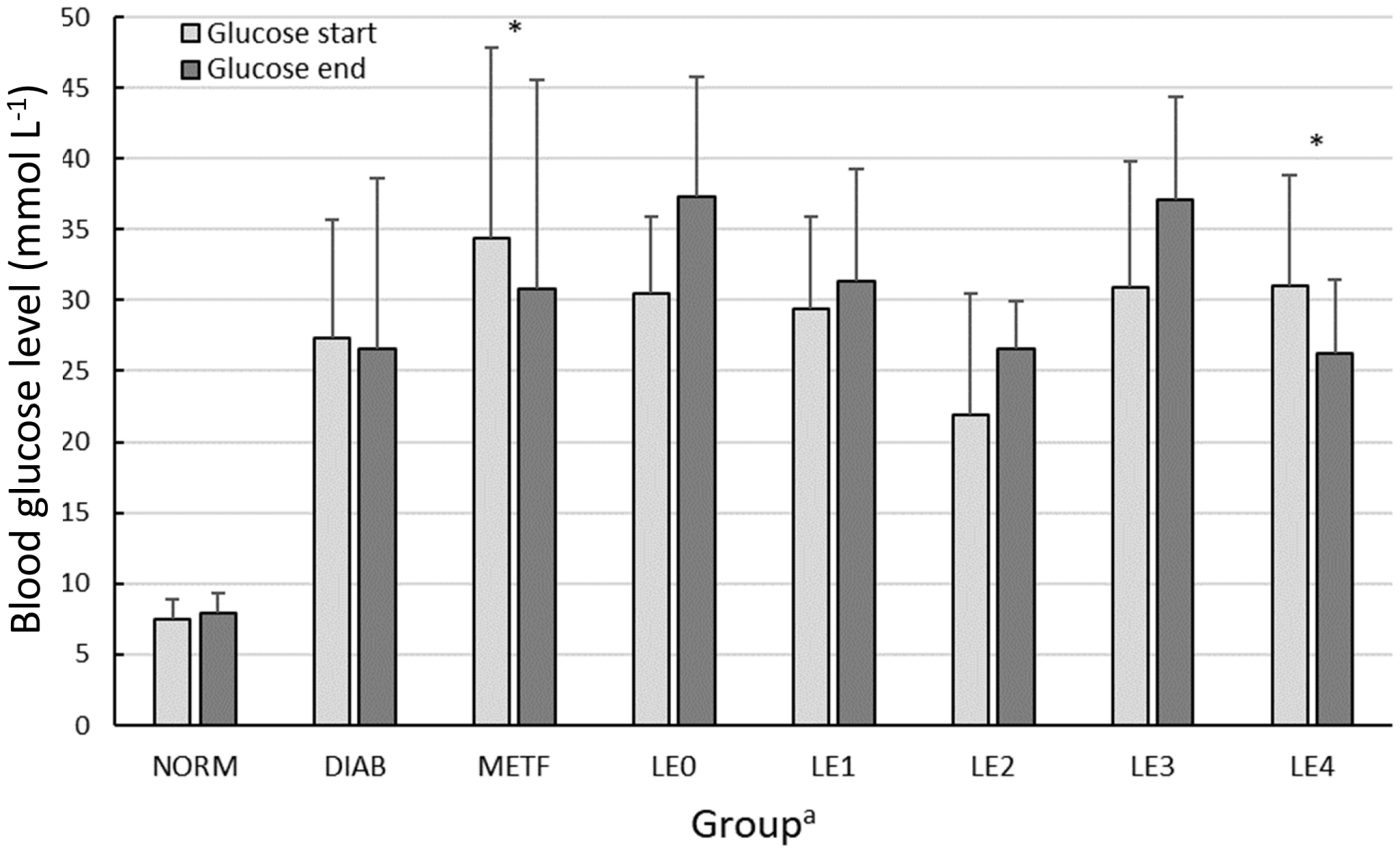
Effect of seven-day treatment with saline, metformin and *Lentinus edodes* extracts on blood glucose level (n = 7; mean ± SD) ^a^blood glucose level of NORM group was excluded from the statistical analysis; *p < 0.05 blood glucose change (glucose start – glucose end) vs LE0 and LE3 group.

The impact of different treatments on diabetes mellitus was assessed by measuring blood glucose levels. As with body weight, glycemia was determined at the beginning of the experiment (Glucose start) and the end of the experiment (Glucose end), as well as the difference between the final and initial values (Glucose change). The results of the group of normoglycemic animals were considered only to confirm the success of the diabetes model but were not included in further statistical processing. Comparing the glycemia values at the beginning of the experiment, which for all diabetic animals represents 48 hours after the application of alloxan, it is observed that there is no statistically significant difference between the groups (**Figure 1**). Analysing the change in glycemia, it can be seen that it is statistically significant when comparing groups treated with metformin (METF) and *L. edodes* of the fourth harvest (LE4) with groups treated with the mushrooms without selenium (LE0) and third harvest (LE3).

**Figure 2:**
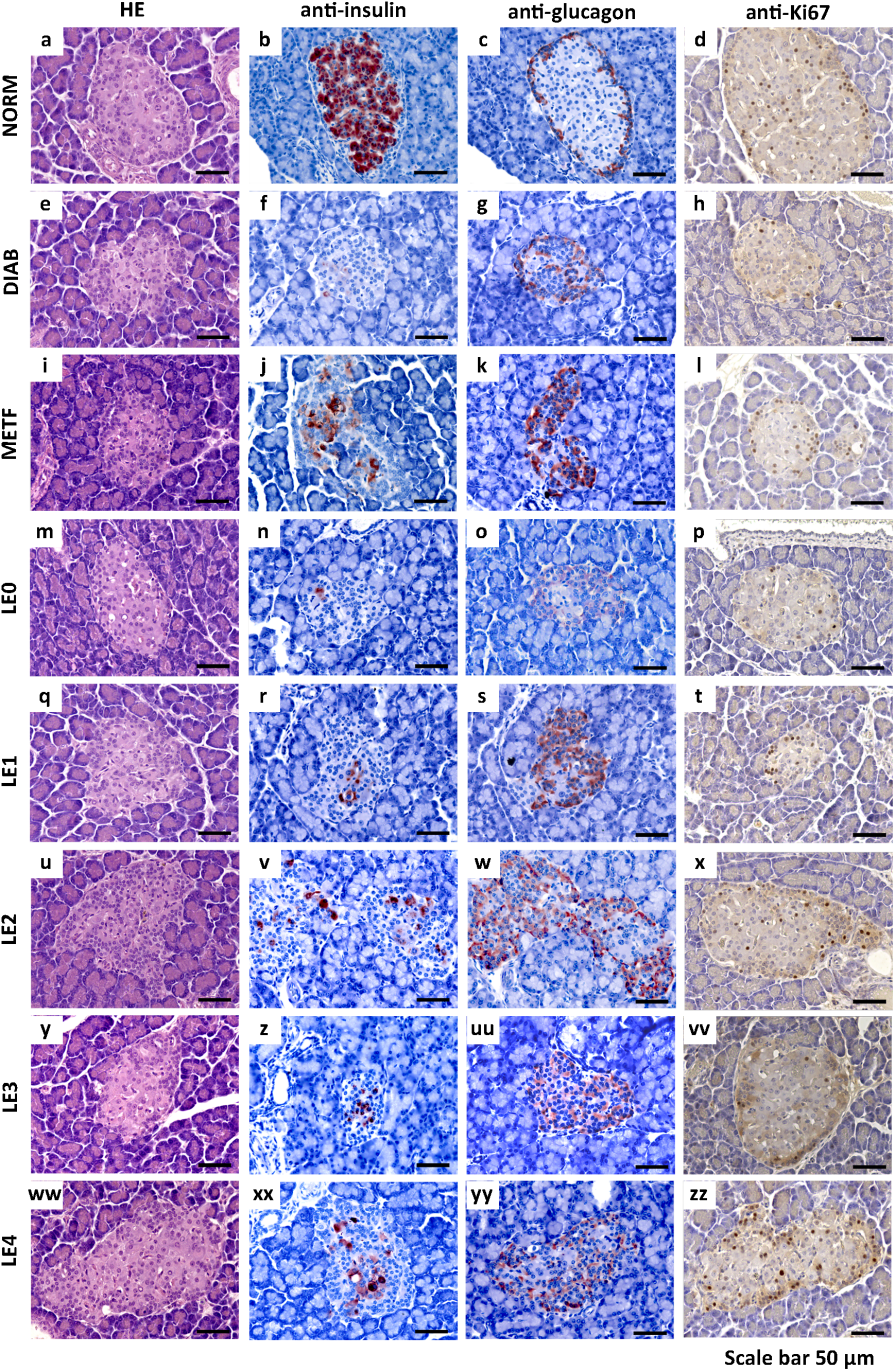
Histochemical and immunohistochemical staining of pancreatic tissue.

Additionally, qualitative histochemical and immunohistochemical analyses of pancreatic tissue in group NORM revealed unique and typical morphological islet characteristics (**Figure 2a**). Beta cells were found in the core of the islets (**Figure 2b**), while alpha cells were located peripherally (**Figure 2c**). Using the proliferative marker Ki67, we identified individual alpha and beta cells in mitosis (**Figure 2d**). Animals in all other groups received alloxan as a diabetes-induced agent, so different islet damage levels were noticed. The most prominent were in the DIAB and METF groups. The islets decreased in size and number, and the regularity of the exocrine-endocrine reticular border (**Figures 2e**, **i**). Generally, the presence of beta cells was reduced or absent (**Figures 2f**, **j**) while alpha cells represented the remaining cell population.

Proliferative changes were mainly noted in the alfa cell population (**Figures h**, **l**). In groups LE0 to LE1, we observed clear damage to islets with varying levels of regenerative changes (**Figures 2m**, **q**, **u**, **y**, **ww**). Unlike the previous groups, a slightly higher number of beta cells could be observed (**Figures 2n**, **r**, **v**, **z**), with particular emphasis on the LE4 group (**Figure 2xx**). The alpha cells increased in all mentioned groups (**Figures 2o**, **s**, **w**, **uu**, **yy**), which was confirmed with the Ki67 proliferative marker (**Figures 2p**, **t**, **x**, **vv**, **zz**).

These radical changes were confirmed using morphometric analysis (**Table 4**). The morphometric analysis of pancreatic tissue focused on the volume density of exocrine (Vvend) and endocrine parts (Vvexo) and the number of alpha (α) and beta (β) cells per islet across different experimental groups. The normoglycemic (NORM) group showed the highest β-cell count, while the diabetic (DIAB) group exhibited a drastic reduction in β-cell numbers, reflecting islet damage. Selenium-enriched *L. edodes* treatments (LE groups) had varying effects. The result of LE4 group was slightly better but not statistically significant compared to other groups. The volume density and α-cell counts remain relatively consistent across groups. However, notable differences are observed in β-cell preservation and overall endocrine health and partly explain the glycemia results.

**Table 4:** Morphometric analysis of pancreatic tissue; (x ± SD).

Group^a^	Volume density of exocrine and endocrine part of pancreatic tissue		Number of α and β cells per islet	
	Vvend	Vvexo	α	β
NORM	2.06 ± 0.11	97.94 ± 0.11	6285 ± 3348	8093 ± 6317
DIAB	1.09 ± 0.10	98.91 ± 0.10	2766 ± 2522	205 ± 159^*^
METF	1.08 ± 0.16	98.92 ± 0.16	2272 ± 1244	948 ± 368
LE0	1.00 ± 0.16^#^	99.00 ± 0.16^#^	1312 ± 1092	216 ± 100^*^
LE1	1.03 ± 0.18	98.97 ± 0.18	1318 ± 507	502 ± 299^*^
LE2	1.23 ± 0.12	98.77 ± 0.12	2449 ± 788	484 ± 180^*^
LE3	1.01 ± 0.16^#^	98.99 ± 0.16^#^	1567 ± 699	464 ± 192^*^
LE4	1.05 ± 0.25	98.95 ± 0.25	2420 ± 1240	1327 ± 355

^a^blood glucose level of NORM group was excluded from the statistical analysis; *p < 0.05 versus METF and LE4 group; ^#^p < 0.05 versus LE2 group.

Many studies have proven that medicinal mushrooms can be helpful in treating diabetes (Sabo et al., 2010; Rašeta et al., 2020; Das et al., 2022). Various mechanisms supporting the anti-diabetic properties of medicinal mushrooms have been described, including the glycemia-lowering effect of polysaccharides, glucose absorption inhibition, α-glucosidase inhibition, and antioxidative effect protecting β-cell damage (Das et al., 2022). The antioxidative effect protecting β-cell damage is one of the probable mechanisms behind the antidiabetic effect of *L. edodes* in our study. Since the induction of diabetes by alloxan administration is based on pro-oxidative activity of alloxan (Rašeta et al., 2020) destroying the functional pancreatic tissue, we can presume that *L. edodes* administration resulted in antioxidant effect thus preserving certain amount of β cells as presented in **Table 4**.

As mentioned above, selenium has well-documented dose-dependent effects on glucose metabolism, often described as non-linear or U-shaped, where both deficiency and excess may exert adverse metabolic consequences, including pro-diabetic activity (Ogawa-Wong et al., 2016; Casanova, 2023). In this context, the stronger antihyperglycemic effect observed in the LE4 group (despite lower selenium content) could reflect a more favorable position within this narrow optimal range of selenium exposure. Importantly, several additional factors may contribute to this finding. First, selenium bioavailability and incorporation into selenoproteins (e.g., glutathione peroxidases) can reach a plateau or become dysregulated at higher intake levels, potentially attenuating beneficial antioxidant and insulin-sensitizing effects (Goldson et al., 2011). On the other hand, the compositional differences between mushroom harvests (e.g., variations in content of polysaccharides, phenolic compounds, and other bioactive compounds) may interact with selenium and modulate the overall metabolic effect, suggesting that selenium content alone does not fully explain the observed outcomes (Łysakowska et al., 2023; da Silva et al., 2024).

Further targeted studies (e.g., controlled selenium dosing and characterization of bioactive compounds) are needed are needed to confirm the underlying mechanism.

#### 3.4.3 Lipid metabolism

**Figure 3:**
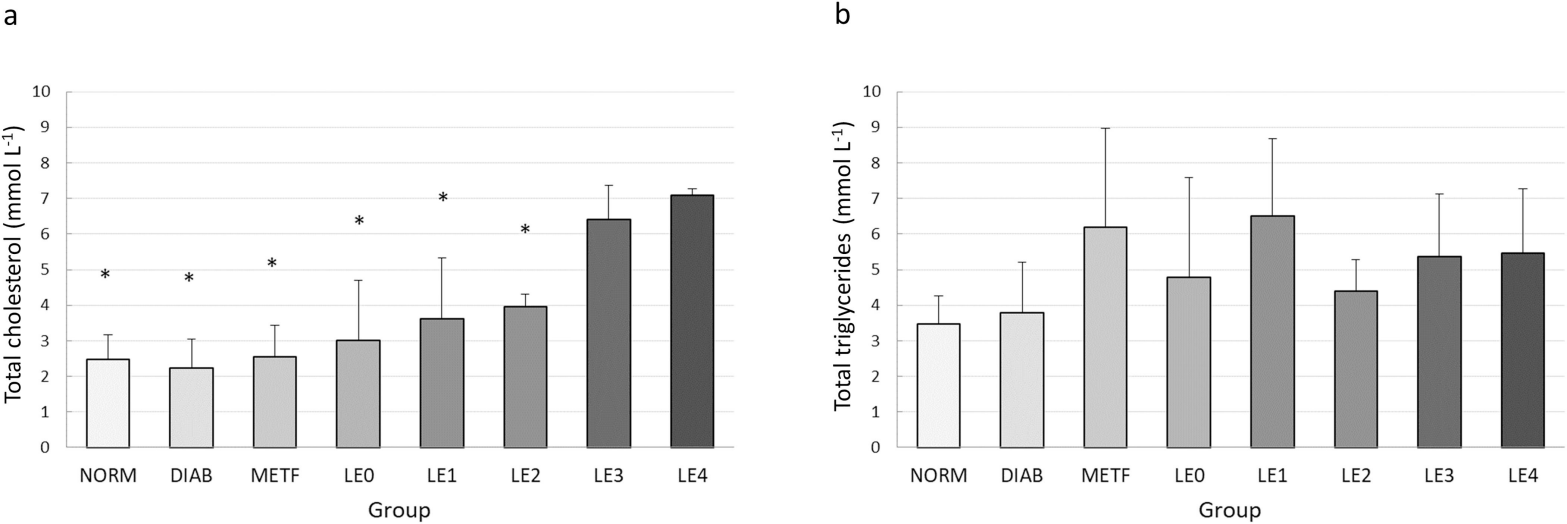
**(a)** Effect of seven-day treatment with saline, metformin and *Lentinus edodes* extracts on blood cholesterol level (n = 7; mean ± SD; mmol L^-1^); **(b)** Effect of seven-day treatment with saline, metformin and *Lentinus edodes* extracts on blood triglycerides level (n = 7; mean ± SD; mmol L^-1^) ^*^p < 0.05 versus LE3 and LE4 group.

In addition to the influence on carbohydrate metabolism, the impact on lipid metabolism was also monitored through serum cholesterol and triglyceride values. Comparing the experimental groups, it is observed that the value of serum cholesterol is statistically significantly higher in the groups treated with *L. edodes* mushroom of the third and fourth harvest (LE3 and LE4) compared to all other groups (**Figure 3a**). Serum triglyceride values varied between groups as well. Although the lowest value was recorded in the group of normoglycemic animals (NORM) and higher in the group treated with *L. edodes* of the fourth harvest (LE4), the statistical significance of these differences was not reached (**Figure 3b**).

Previous animal and human studies have indeed reported a positive association between selenium exposure and circulating lipid levels, including total cholesterol and triglycerides (Pinto et al., 2012; Christensen et al., 2015; Nie et al., 2023), which is consistent with our findings. The underlying mechanisms may involve selenium-dependent modulation of hepatic lipid metabolism. In particular, selenoproteins such as selenoprotein P and glutathione peroxidases play an important role in redox regulation and liver function, which are closely linked to lipid synthesis, lipoprotein assembly, and lipid clearance (Mita et al., 2017). Increased selenium availability may alter these pathways, potentially promoting hepatic lipoprotein production or affecting lipid turnover. In other words, selenium exhibits a narrow physiological range, and higher exposure levels may lead to metabolic effects that differ from those observed at optimal intake, similar to glucose metabolism. In this context, increases in circulating lipids could reflect a shift toward altered hepatic lipid handling at higher selenium levels. Evidence suggests a potential U-shaped relationship between selenium status and lipid profiles, such that both insufficient and excessive selenium levels may contribute to adverse lipid outcomes (Mita et al., 2017). Additionally, as mentioned above L. edodes represents a complex biological matrix, and differences in composition between harvests (e.g., levels of sterols, polysaccharides, or other bioactive compounds) may contribute to the observed changes in lipid parameters, potentially interacting with selenium and modulating its metabolic effect (Chen et al., 2015; Zhang et al., 2020; Łysakowska et al., 2023; da Silva et al., 2024).

Therefore, our findings suggest that selenium enrichment of *L. edodes* may influence lipid metabolism, and that these effects should be considered when evaluating its overall metabolic impact.

#### 3.4.4 Selenoproteins

**Figure 4:**
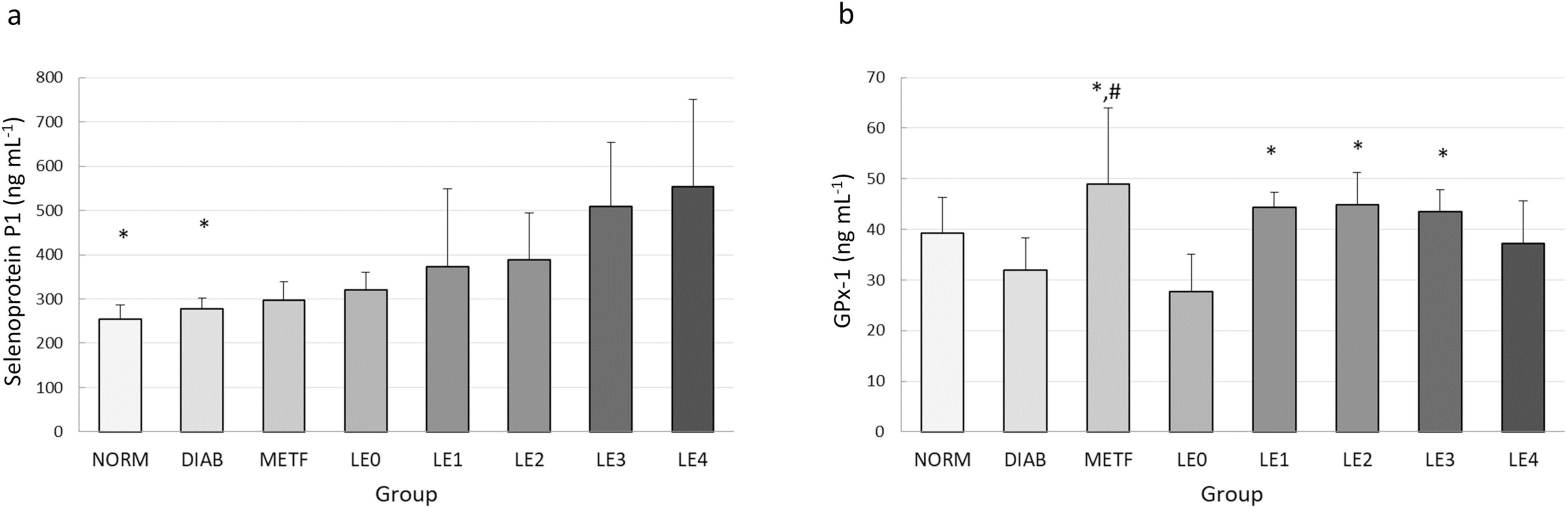
**(a)** Effect of seven-day treatment with saline, metformin and *Lentinus edodes* extracts on selenoprotein P1 serum level (n = 7; mean ± SD; ng mL^-1^) ^*^p < 0.05 versus LE4 group **(b)** Effect of seven-day treatment with saline, metformin and *Lentinus edodes* extracts on glutathione peroxidase 1 (GPx-1) serum level (n = 7; mean ± SD; ng mL^-1^) *p<0.05 vs LE0; ^#^p<0.05 vs DIAB.

The effect of a seven-day treatment with *L. edodes* mushroom on selenoproteins was assessed by the concentration of selenoprotein P1 and glutathione peroxidase 1 (GPx-1) in the serum of animals. In **Figure 4a**, it is observed that the values of selenoprotein P1 of all groups treated with selenium-enriched mushroom (LE1-4) are higher than the values of control groups of animals (NORM, DIAB, METF, LE0). However, a statistically significant difference exists only when comparing the group of *L. edodes* fourth harvest (LE4) with the group of normoglycemic (NORM) and diabetic animals (DIAB) treated with saline. Additionally, a correlation was made between the values of selenoprotein P1 and the values of body mass, blood sugar, total cholesterol and triglycerides. A strong correlation was found between the value of selenoprotein P1 and total cholesterol (ρ = 0.622; p < 0.001).

Regarding GPx-1 values, the findings are more complex. The highest values were recorded in the group of diabetic animals treated with metformin (METF), while the lowest were in the group receiving *L. edodes* without selenium (LE0). All groups of animals that received selenium-enriched mushrooms had higher GPx-1 values compared to the control group of diabetic animals (DIAB) and the group of mushrooms without selenium (LE0). Statistical significance of the difference was reached when comparing groups treated with metformin or mushroom from the first to third harvest and those treated with selenium-free mushroom (**Figure 4b**).

Contemporary research points to the fact that selenoproteins do not reflect simple selenium intake but are involved in the etiology of systemic diseases and disorders such as diabetes mellitus, dyslipidemia, obesity, insulin resistance, fatty liver and metabolic syndrome (Ogawa-Wong et al., 2016; Tinkov et al., 2020; Casanova, 2023). Despite a large number of studies, it is still not entirely clear whether increased intake of selenium and the consequent increase in selenoproteins can prevent or promote the occurrence of the previously mentioned diseases and disorders. Also, there are inevitable disagreements in the definition of increased intake of selenium and the recommended daily intake, which altogether impact the recommendations regarding the safety of selenium use (Vinceti et al., 2022; Casanova, 2023). It is essential to point out that the chemical form of applied selenium also plays a role in positive and adverse health effects. Although it is emphasized that organic selenium has more positive and less adverse effects on health, some studies show that inorganic selenium can also have favourable metabolic effects by increasing the expression of genes for selenoprotein P1 and GPx-1 (El-Magd et al., 2022; Vinceti et al., 2022).

Although not all the physiological functions of selenoproteins are known, it is believed that selenoprotein P1 and GPx-1 represent antioxidant enzymes and that increasing their concentration in the blood aims to reduce the consequences of oxidative stress (Maseko et al., 2014; El-Magd et al., 2022; Vinceti et al., 2022). As mentioned earlier, all groups of animals treated with *L. edodes* enriched with selenium had higher values of selenoprotein P1 than all other groups. GPx-1 values were higher in all groups treated with selenium-enriched *L. edodes* compared to the selenium-free mushroom group. Interestingly, the treatment with mushrooms of the first and second harvest, which have a higher selenium content, showed a similar result to the treatment with metformin regarding GPx-1 (**Figure 4b**). Thus, we believe that the increased concentrations of both examined selenoproteins can be related to the defence against oxidative stress evident in the alloxan diabetes model (Popović et al., 2010; Rašeta et al., 2020). Finally, the result that in the group of animals that received the mushroom of the fourth harvest, with the lowest content of selenium, the highest concentration of selenoprotein P1 was recorded, and its correlation with the concentration of total cholesterol is surprising and requires further research. Clinical studies have recorded an increased concentration of selenoproteins in chronic carbohydrate and fat metabolism disorders. Still, it is not known whether the increase is a cause or a consequence (Vinceti et al., 2022; Casanova, 2023). Therefore, we think a different animal model of diabetes and a more prolonged exposure to increased selenium intake may provide a more adequate answer.

## 4 Conclusion

This study demonstrated that exogenous selenium application effectively promoted selenium biofortification in *L. edodes*. Additionally, the biofortified mushrooms were associated with increased circulating levels of selenoprotein P and GPx-1, underscoring their potential as functional foods with antioxidant benefits. The results suggest that selenium-enriched L. edodes extracts may possess antidiabetic potential; however, the most significant glucose-lowering effect was observed in the LE4 group, indicating that this activity may depend on the specific characteristics and selenium content of the extract. These findings emphasise selenium-enriched mushrooms’ nutritional potential in addressing oxidative stress and metabolic disorders. Future research should focus on optimising selenium enrichment methods and exploring long-term health benefits in preclinical and clinical settings to harness their potential fully.

## Data Availability

The raw data supporting the conclusions of this article will be made available by the authors, without undue reservation.
